# Supplementary material for: Lack of androgen receptor SUMOylation results in male infertility due to epididymal dysfunction
Source: Nat Commun. 2019 Feb 15;10:777. doi: 10.1038/s41467-019-08730-z (PMC6377611; doi:10.1038/s41467-019-08730-z)
Supplement: Supplementary file 1 — Supplementary Information [file 41467_2019_8730_MOESM1_ESM.pdf]

Supplementary information

Lack of androgen receptor SUMOylation results in male infertility due to epididymal dysfunction

Zhang et al.

Supplementary Figure 1

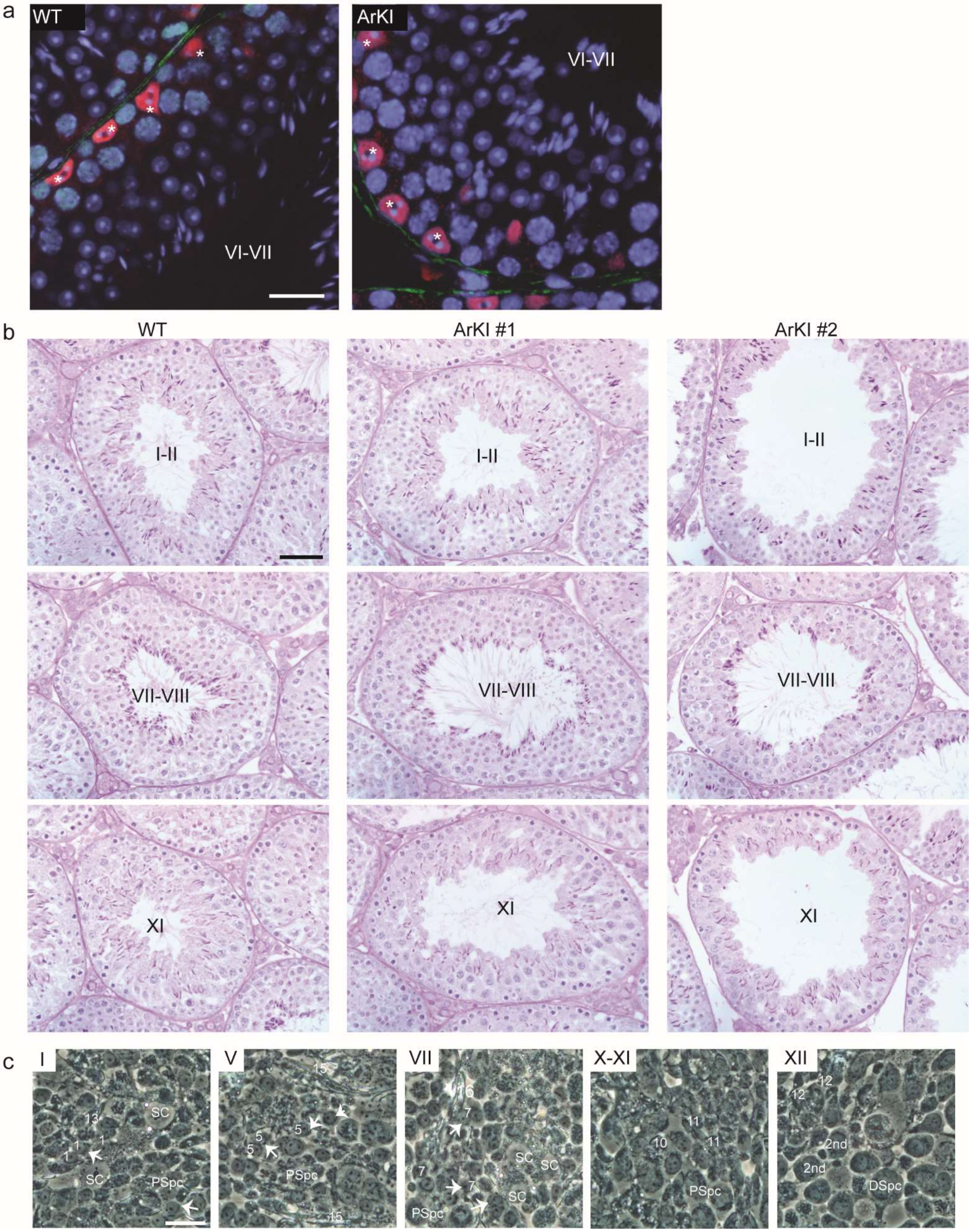

Supplementary Figure 1. Testicular histology is not affected by AR SUMOylation **a** Formalin-fixed paraffin-embedded testis sections (stage VI-VII) of 3 months old wild type (WT) and ArKI mutant mice were immunostained with antibodies against AR (red) and smooth muscle actin (green). Nuclei were stained with DAPI. AR localized in Sertoli cell nuclei (asterisks) in both control and mutant mice. Scale bar: 20 µm. **b** Bouin-fixed paraffin-embedded testis sections of 6-7 months old WT and ArKI mice were stained with Periodic acid–Schiff (PAS). Example images of three different stages of the seminiferous epithelial cycle are shown (I-II, VII-VIII and XI). In around half of the ArKI testes the lumen of the seminiferous tubule appeared expanded as exemplified by the ArKI mouse #2. Scale bar 50 µm. **c** Phase contrast microscopy analysis of the stage-specific squash preparations of ArKI seminiferous tubules. Developing acrosomes in round spermatids are indicated with arrows. The step of spermatid differentiation is indicated with numbers 1-16. SC, Sertoli cell; PSpc, pachytene spermatocyte; DSpc, diplotene spermatocyte; 2nd Spc, secondary spermatocyte. Scale bar: 20 µm.

Supplementary Figure 2

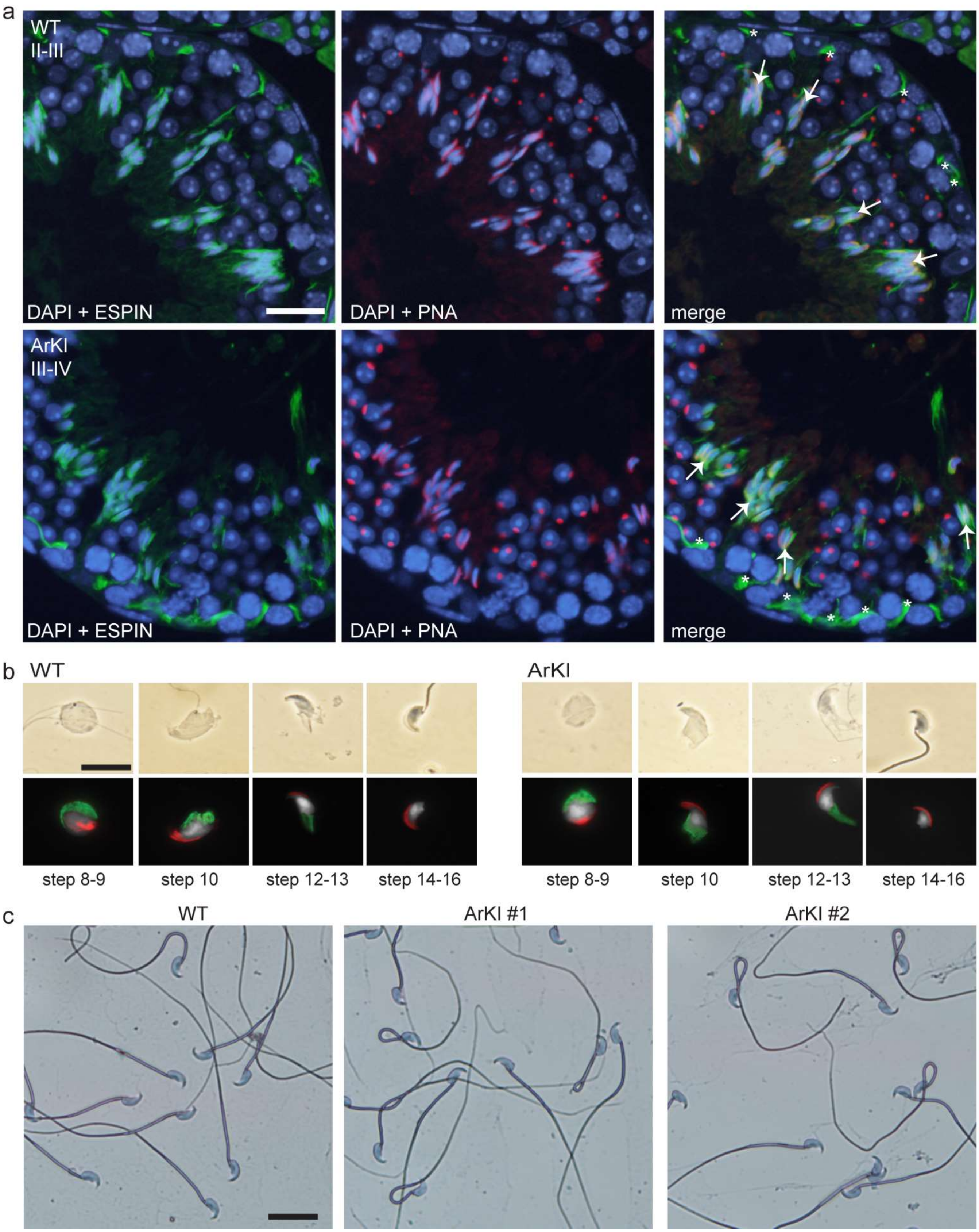

Supplementary Figure 2. Spermatogenesis and spermatozoa appear normal in ArKI mice. **a** Formalin-fixed paraffin-embedded testis sections of 3 months old WT and ArKI mice were immunostained with anti-ESPIN antibody (green). Acrosomes were stained with PNA-lectin (red) and nuclei were stained with DAPI (blue). ESPIN signal is detected in the cell-cell junctions between two adjacent Sertoli cells (blood-testis barrier, asterisks) and between Sertoli cells and elongating spermatids (apical ectoplasmic specializations, arrows). Scale bar 20 µm. **b** Stage-specific drying down preparations from 6 months old WT and ArKI mice were immunostained with anti-TUBULIN (green) to visualize the microtubular manchette structure in elongating spermatids. Acrosomes were stained with PNA-lectin (red) and nuclei were stained with DAPI (grey). The sept of spermatid differentiation is indicated with numbers 8-16. Scale bar 10 µm. **c** Spermatozoa were isolated from cauda epididymidis of 6 months old WT and ArKI mice and stained with hematoxylin. Scale bar 10 µm.

Supplementary Figure 3

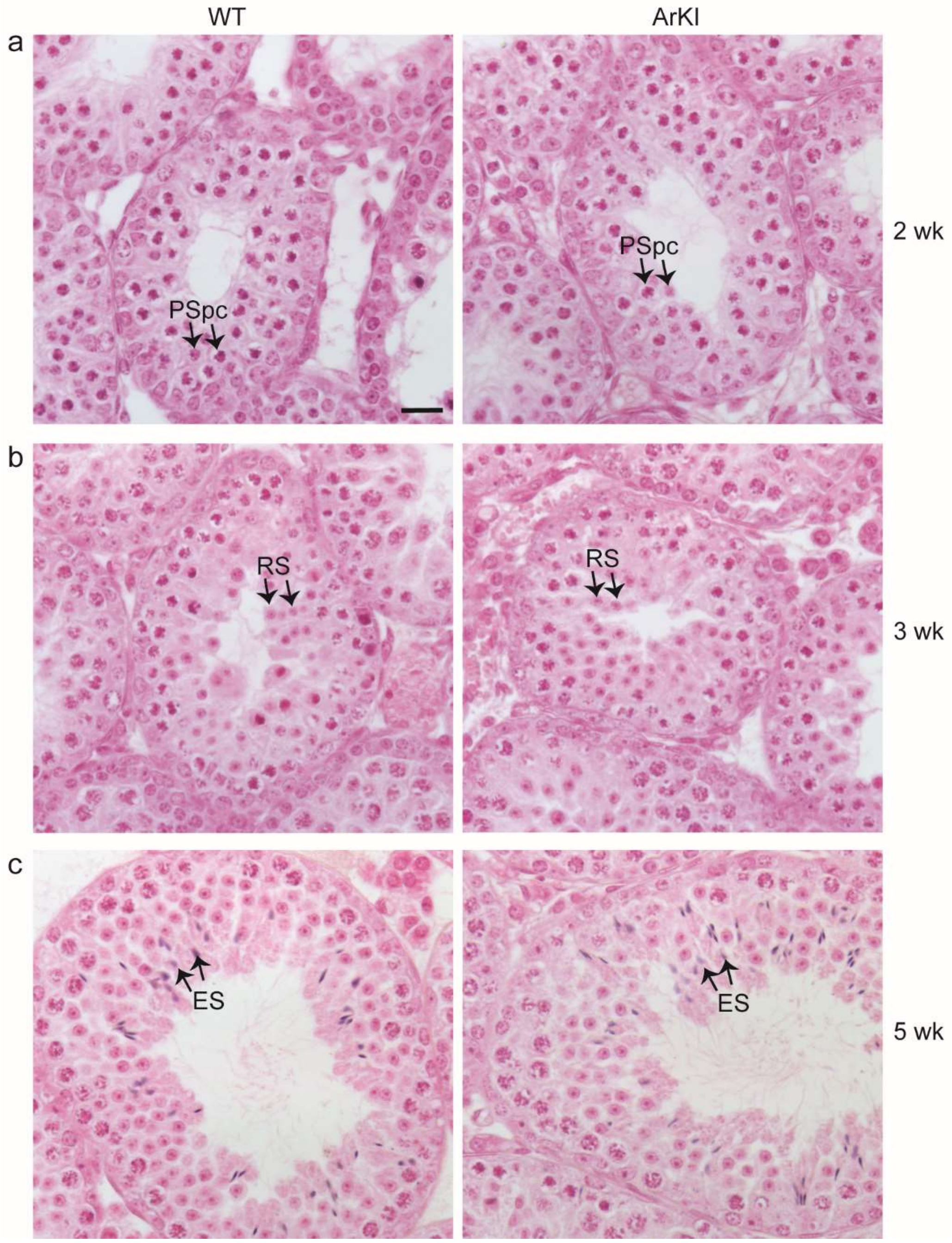

Supplementary Figure 3. Testis histology of juvenile mice. Bouin-fixed paraffin-embedded testis sections of **a** 2 weeks old, **b** 3 weeks old, and **c** 5 weeks old WT and ArKI mice were stained with hematoxylin and eosin (HE). In both WT and ArKI testes, the first wave of spermatogenesis had progressed until pachytene spermatocyte (PSpc) phase at 2 weeks and round spermatid (RS) phase at 3 weeks. At 5 weeks, the full spermatogenesis with condensed elongating spermatids (ES) was observed. Black arrows indicate examples of each cell type. Scale bar 20 µm.

Supplementary Figure 4

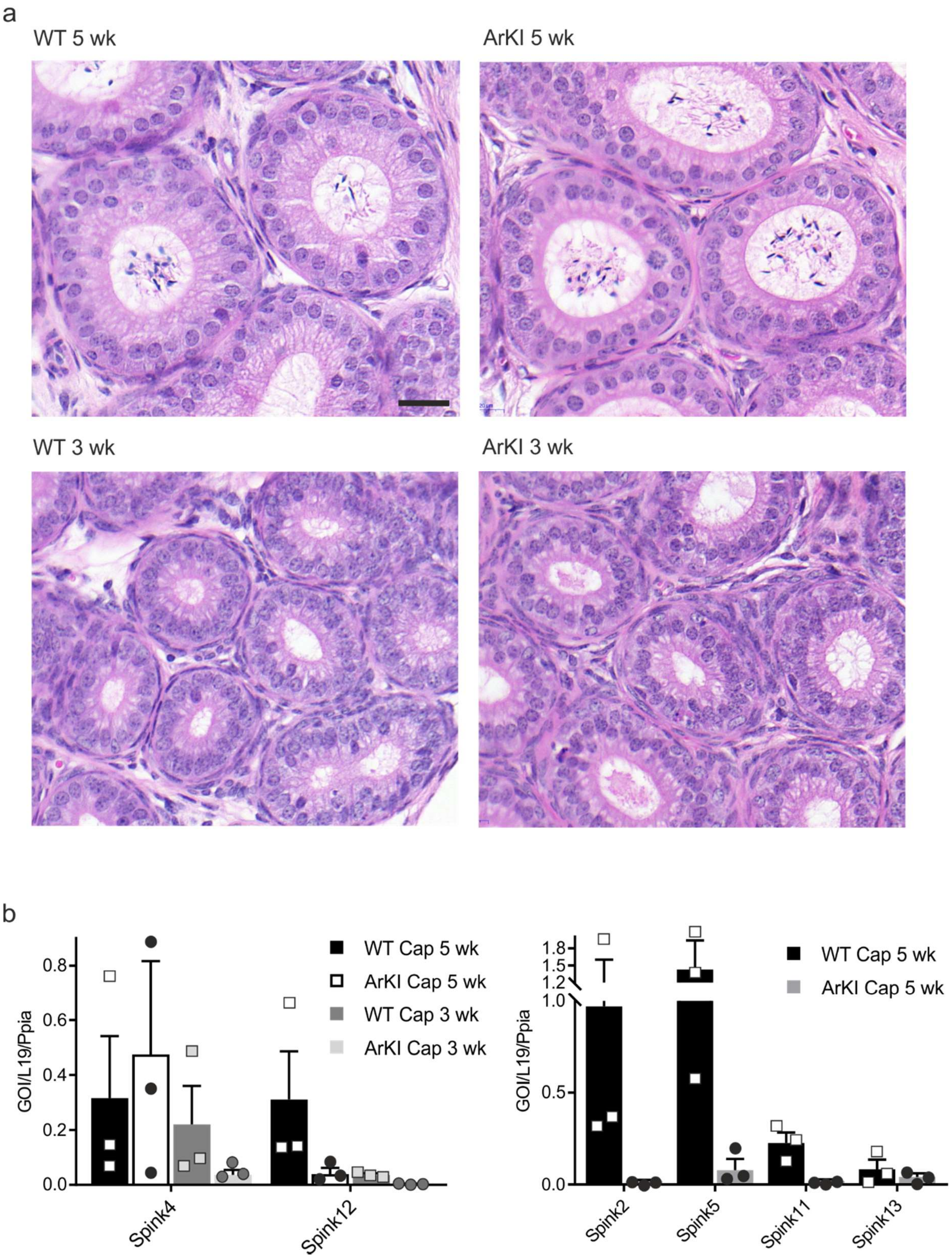

Supplementary Figure 4. AR SUMOylation affects epididymal gene expression but not histology of juvenile mice. **a** Epididymal histology of five and three weeks old wild type (WT) and ArKI caput. Scale bar 20 µm. **b** Relative expression (mean ± SEM, 3 WT and 3 ArKI samples) of *Spink2*, *Spink4*, *Spink5*, *Spink11*, *Spink12* and *Spink13* mRNA in WT and ArKI Cap epididymides at the age of 5 and 3 weeks by RT-qPCR normalized to *L19* and *Ppia* expression.

Supplementary Figure 5

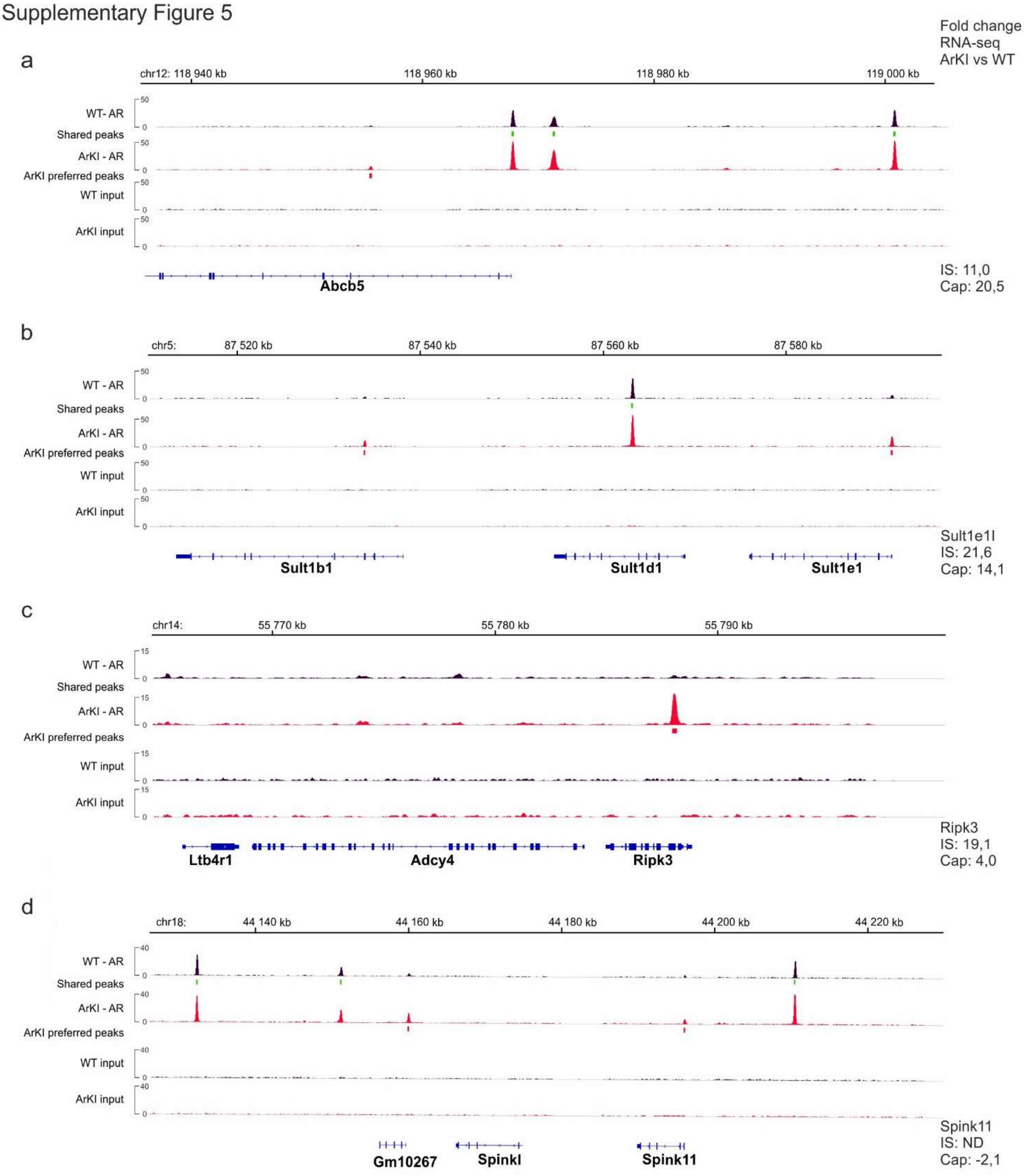

Supplementary Figure 5. Epididymal AR ChIP-seq. Examples of up-regulated genes with a stronger binding of the mutant AR to the shared ARBs compared to WT AR (**a** and **b**), or only mutant AR binding (**c**). **d** example of AR binding in proximity of down-regulated genes. ChIP-seq track examples of *in vivo* AR loading and input controls at differentially expressed loci in the epididymis of WT and that of ArKI mice (WT, black; ARKI, red). The green and red bars indicate the positions of WT- and ARKI-shared ARBs and ArKI-preferred ARBs, respectively. IS, initial segment; Cap, caput.

Supplementary Figure 6

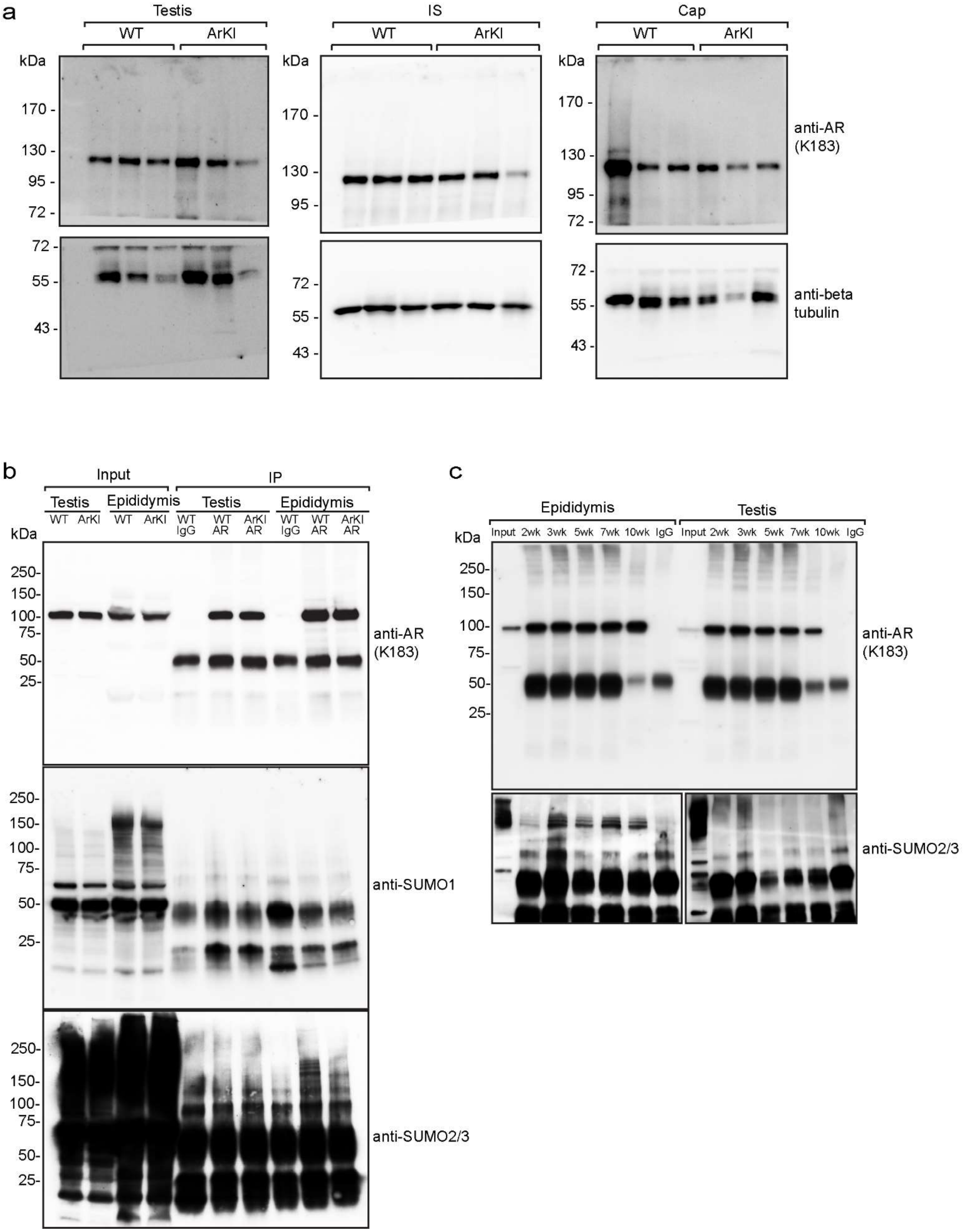

Supplementary Figure 6. Uncropped membranes. **a** Western blotting of AR in ArKI and WT epididymis and testis (from Figure 1d). **b** AR, SUMO-1 and SUMO-2/3 immunoblots from the input lysate samples, anti-AR-immunoprecipites and IgG-immunoprecipites of ArKI and WT mice (From Figure 5a). **c** AR, and SUMO-2/3 immunoblots from the input lysate samples, anti-AR-immunoprecipites and IgG-immunoprecipites from juvenile animals (from Figure 5b).

Supplementary Table 1. Serum gonadotropin and serum, intraepididymal and intratesticular steroid concentrations in adult WT and ArKI males.

	**LH (ng/ml)**	**FSH (ng/ml)**	**A-dione (pg/ml or pg/g)**	**T (pg/ml or pg/g)**	**DHT (pg/ml or pg/g)**
Serum					
WT	0.39 (±0.28)	44.16 (±1.74)	26 (±7)	695 (±307)*	30 (±8)
ArKI	0.19 (±0.06)	53.81 (±5.72)	44 (±8)	1178 (±307)	43 (±5)
Intraepididymal					
WT			279 (±113)#	2544 (±1464)	19254 (±2974)
ArKI			115 (±28)	1514 (±226)	18556 (±774)
Intratesticular					
WT			51218 (±4149)	106063 (±12856)	924 (±132)
ArKI			43114 (±6261)	119632 (±20197)	596 (±82)

Values are given as mean ± SEM.

* Two-tailed Student's t-test, t=1.114, df=14, p = 0.2842

\# Two-tailed Mann-Whitney test, p = 0.1556

Supplementary Table 2. *De novo* motif analysis of ArKI-preferred AR chromatin-binding sites (ARBs) and that of WT- and ArKI-shared ARBs listing the 10 most enriched transcription factor motifs within the both ARB categories.

ArKI preferred ARBs with motif (8 518)		p-value	% of target/ % of back-ground	Shared ARBs with motif (17 648)		p-value	% of target/ % of back-ground
ARE	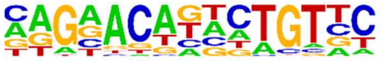	1.00E -2759	84.8/ 26.6	ARE	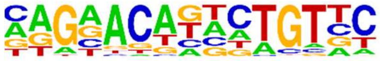	1.00E - 5147	83.0/ 27.5
AP-2	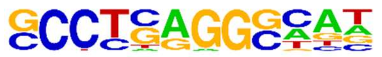	1.00E -177	11.3/ 4.0	Egr1	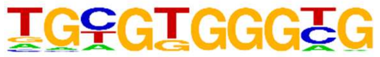	1.00E- 283	12.6/ 5.5
Nkx6.1	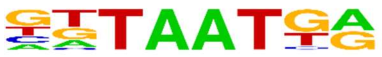	1.00E -131	45.6/ 32.9	Nkx6.1	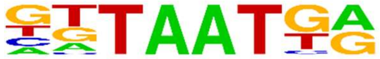	1.00E- 240	37.0/ 25.7
Lhx2	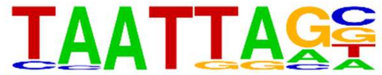	1.00E -92	21.1/ 13.1	PAX5	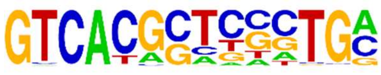	1.00E- 231	8.0/ 3.2
NF1	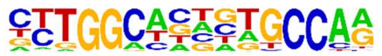	1.00E -81	6.3/ 2.5	NF1	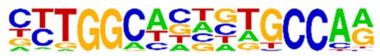	1.00E- 211	8.3/ 3.3
PAX5	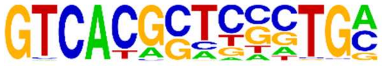	1.00E -75	6.2/ 2.5	Lhx2	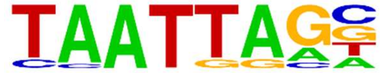	1.00E- 143	16.4/ 10.1
Isl1	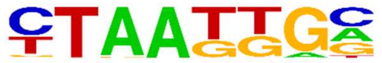	1.00E -74	31.16/ 22.5	AP-2	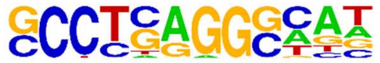	1.00E- 135	10.1/ 5.9
ELF5	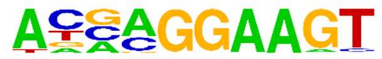	1.00E -72	12.9/ 7.3	Tcf4	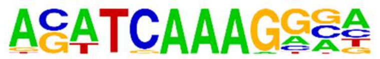	1.00E- 132	8.7/ 4.5
Nanog	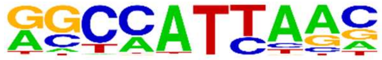	1.00E -69	56.5/ 46.6	Isl1	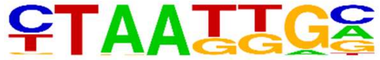	1.00E- 116	27.0/ 19.8
ZNF675	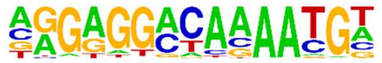	1.00E -59	3.5/ 1.2	ZNF189	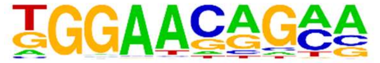	1.00E- 94	14.8/ 9.8

Supplementary Table 3. TMM normalized RPKM (Reads Per Kilobase of exon per Million reads mapped) expression values of SUMOylation pathway genes in WT and ArKI initial segment (IS), Caput (Cap) epididymidis and testis (Te) from RNA-seq.

Gene ID	Entrez ID	WT IS	ArKI IS	WT Cap	ArKI Cap	WT Te	ArKI Te
Sumo1	22218	17.4	17.1	17.1	18.0	23.6	25.1
Sumo2	170930	0.2	0.4	0.4	0.3	0.5	0.4
Sumo3	20610	49.3	46.4	41.0	43.7	22.3	23.4
Sae1	56459	58.1	59.6	35.5	34.4	73.4	76.3
Uba2 *	50995	39.7	40.5	48.6	45.9	80.7	81.1
Ube2i #	22196	4.7	4.5	5.4	4.9	7.9	7.6
Pias1	56469	17.4	21.8	28.0	38.2	83.2	85.3
Pias2	17344	14.2	17.0	13.1	13.2	158.7	157.9
Pias3	229615	9.0	7.9	7.7	8.8	3.9	3.6
Pias4	59004	9.5	8.3	11.2	9.6	116.0	113.3
Senp1	223870	9.1	8.9	6.4	5.9	30.2	28.7
Senp2	75826	13.5	13.1	14.2	12.8	101.3	99.8
Senp3	80886	20.2	19.9	22.4	21.7	35.2	36.1
Senp5	320213	10.0	9.8	9.4	8.9	6.8	6.5
Senp6	215351	27.9	29.1	22.0	22.3	29.7	29.7
Senp7	66315	5.2	5.5	5.4	5.1	3.0	2.8
Desi1	28075	19.8	15.3	13.6	12.5	231.4	239.1
Desi2	78825	9.7	9.4	5.6	6.5	9.1	9.4

* also known as Sae2

# also known as Ubc9

Supplementary Table 4. Genes whose expression is significantly changed in both SPARKI and ArKI caput epididymidis.

Gene ID	EntrezID	ArKI FC	SPARKI FC	Ref.
Rarres1	109222	-3.4	-2.2	23
Pkd2l1	329064	-5.8	3.6	23
Dmrtc1a	70887	-3.1	-1.5	23
Mcoln3	171166	-6.2	-1.6	23
Capn3	12335	-4.2	-1.2	23
Alox5	11689	-4.2	-0.8	23
Cav3	12391	-4.2	-2.2	23
Sema3g	218877	-2.8	0.7	23
C1qtnf3	81799	-4.9	-1.2	23
Kcna1	16485	-2.8	4.11	22

Supplementary Table 5. Primers used in cloning, mutagenesis, screening, PCR and qPCR analyses

Gene	Forward primer sequence	Tm (℃)	Used in
Primer A	TGCCCCTTCATTCAATACCCTTGGACTAAATCATTCAAAAGC TTT GGTAG	76	Cloning
Primer B	AGACTAGACAGCAGGAGGGAACTGTAAAATGAATAGCCACA TAGC AGAAC	74	Cloning
Primer C	TTGCATTTTGTGGGATCTAGGGACTCCCGCCCAATAGAACA A TCGGAAGGATCC	83	Cloning
Primer D	GGGAATTGGAGGGGAATCTCAAAGAGCACGTATTAGCACCC CGGAGCGCA	84	Cloning
AR mutS1	CCACACGCCCGTATCAGGCTCGAGAACCCATTGGAC	78.9	Mutagenesis
AR mutAs1	GTCCAATGGGTTCTCGAGCCTGATACGGGCGTGTGG	78.9	Mutagenesis
AR mutSe2	CCCAATTGTGTCAGAAGTGAAATGGGACC	65.8	Mutagenesis
AR mutAs2	GGTCCCATTTCACTTCTGACACAATTGGG	65.8	Mutagenesis
AR 5arm	CAGTGTTCTCTTTCACTGTTGGTCTACA	57	Screening
Neo2 As	CGGTGGATGTGGAATGTGTGCGA	64	Screening
Neo Se1	CCTCGTGCTTTACGGTATCG	52	Screening
AR 3arm	GGAGTGGGGGTGACAAAATA	51	Screening
AR5ArmSe	GGCTGACAAGCCAGGAGAGTGA	58	Screening
AR3armAs	CCCATGCAATTGATTTAAGTTTGGAACT	60.5	Screening
ArKI WT F	ATCTTGTGAAATGTTCCGCC	51.5	Genotyping
ArKI mut F	AGCCCTATAGTGAGTCGTATTA	46	Genotyping
ArKI R	CCCATGCAATTGATTTAAGTTTGGAACT	60.5	Genotyping
Ar F	GTCTCCGGAAATGTTATGAA	47	RT-qPCR
Ar R	AAGCTGCCTCTCTCCAAG	46.5	RT-qPCR
Spink2 F	CATGAGACTCTCGACTCTTCCG	53.1	RT-qPCR
Spink2 R	CGCACACAGGGTTGAGGTT	53.1	RT-qPCR
Spink4 F	TATGCATCTGTGGCTGGTCA	52.1	RT-qPCR
Spink4 R	GCTCAGCCATGTGCTCACAG	54	RT-qPCR
Spink5 F	GTCCCAAAGGCAACAATTCTTC	54.3	RT-qPCR
Spink5 R	TGATCTTGGTAGGTGCTCCTTG	53.4	RT-qPCR
Spink11 F	ATGTGTAACGAGGTAGAAGA	50	RT-qPCR
Spink11 R	GGAAGAAGTACCAAAGTCAG	50.3	RT-qPCR
Spink12 F	TTCAACGCCCCTAACATTTCTG	55.7	RT-qPCR
Spink12 R	AATGCACAGCGGTTTTGGTAG	54.4	RT-qPCR
Spink13 F	ATGCTGCTCTCCCTCGTACTT	52.3	RT-qPCR
Spink13 R	TCACATCTGGACAGTTAGCTTCA	51.7	RT-qPCR
Rhox5 F	ACTCGGAAGAACAGCATGATG	55	RT-qPCR
Rhox5 R	CCCTGGTGCCACTATCCTT	56.8	RT-qPCR
L19 F	GGACAGAGTCTTGATGATCTC	52.1	RT-qPCR
L19 R	CTGAAGGTCAAAGGGAATGTG	53.6	RT-qPCR
Ppia F	CATCCTAAAGCATACAGGTCCTG	55.1	RT-qPCR
Ppia R	TCCATGGCTTCCACAATGTT	54.8	RT-qPCR
