# Supplementary material for: Lack of androgen receptor SUMOylation results in male infertility due to epididymal dysfunction
Source: Nat Commun. 2019 Feb 15;10:777. doi: 10.1038/s41467-019-08730-z (PMC6377611; doi:10.1038/s41467-019-08730-z)
Supplement: Supplementary file 3 — Description of Additional Supplementary Files [file 41467_2019_8730_MOESM3_ESM.pdf]

Description of Additional Supplementary Files

File Name: Supplementary Data 1
Description: A large Excel Table containing three separate sheets.

Sheet 1: Differentially expressed androgen-regulated genes in ArKI IS vs. WT IS (FDR < 0.05 and FC ≥2). Mean values are TMM normalized counts represented as RPKM values.
Sheet 2: Differentially expressed androgen-regulated genes in ArKI caput vs. WT caput (FDR below 0.05 and FC ≥ 2). Mean values are TMM normalized counts represented as RPKM values.
Sheet 3: Differentially expressed genes ArKI vs WT testis (FDR 0.2 and FC > 2). Mean values are TMM normalized counts represented as RPKM values.
