# Supplementary material for: Lack of androgen receptor SUMOylation results in male infertility due to epididymal dysfunction
Source: Nat Commun. 2019 Feb 15;10:777. doi: 10.1038/s41467-019-08730-z (PMC6377611; doi:10.1038/s41467-019-08730-z)
Supplement: Supplementary file 5 — Reporting Summary [file 41467_2019_8730_MOESM5_ESM.pdf]

nature research

Corresponding author(s): Jorma J. Palvimo

Last updated by author(s): Jan 9, 2019

Reporting Summary

Nature Research wishes to improve the reproducibility of the work that we publish. This form provides structure for consistency and transparency in reporting. For further information on Nature Research policies, see Authors & Referees and the Editorial Policy Checklist.

Statistics

For all statistical analyses, confirm that the following items are present in the figure legend, table legend, main text, or Methods section.

n/a	Confirmed	
☐	☒	The exact sample size (*n*) for each experimental group/condition, given as a discrete number and unit of measurement
☒	☐	A statement on whether measurements were taken from distinct samples or whether the same sample was measured repeatedly
☐	☒	The statistical test(s) used AND whether they are one- or two-sided<br>*Only common tests should be described solely by name; describe more complex techniques in the Methods section.*
☒	☐	A description of all covariates tested
☐	☒	A description of any assumptions or corrections, such as tests of normality and adjustment for multiple comparisons
☐	☒	A full description of the statistical parameters including central tendency (e.g. means) or other basic estimates (e.g. regression coefficient) AND variation (e.g. standard deviation) or associated estimates of uncertainty (e.g. confidence intervals)
☒	☐	For null hypothesis testing, the test statistic (e.g. *F*, *t*, *r*) with confidence intervals, effect sizes, degrees of freedom and *P* value noted<br>*Give P values as exact values whenever suitable.*
☒	☐	For Bayesian analysis, information on the choice of priors and Markov chain Monte Carlo settings
☒	☐	For hierarchical and complex designs, identification of the appropriate level for tests and full reporting of outcomes
☒	☐	Estimates of effect sizes (e.g. Cohen's *d*, Pearson's *r*), indicating how they were calculated

Our web collection on statistics for biologists contains articles on many of the points above.

Software and code

Policy information about availability of computer code

Data collection	No software were used.
Data analysis	For RNA-seq data analyses, the quality of the sequenced reads was checked using FastQC tool version 0.11.4.<br>Star v2.5.2b was used to align the reads.<br>The number of uniquely mapped reads associated with each gene was counted using subreads package.<br>The downstream analysis of the data was performed using R version 3.3 and its corresponding Bioconductor module 3.3. Specifically, the following R packages were used:<br>The count data were normalized for library size using the Trimmed Mean of M-values (TMM) method implemented in edgeR package.<br>For statistical testing the data were further transformed using the voom approach in the limma package.<br>Differential expression analysis was carried out using the ROTS package.<br>Enrichment analysis of the differentially expressed genes was performed with topGO, GOstats and GO.db packagee.<br>The hierarchical clustering of RNA-seq data was performed using the pheatmap package.<br>The software used for ChIP-seq analyses is described later in ChIP-seq -section of this form. For rest of the statistical analyses GraphPad Prism 7.0 software (GraphPad Software, USA) was used.

For manuscripts utilizing custom algorithms or software that are central to the research but not yet described in published literature, software must be made available to editors/reviewers. We strongly encourage code deposition in a community repository (e.g. GitHub). See the Nature Research guidelines for submitting code & software for further information.

Data

Policy information about availability of data

All manuscripts must include a data availability statement. This statement should provide the following information, where applicable:

- Accession codes, unique identifiers, or web links for publicly available datasets
- A list of figures that have associated raw data
- A description of any restrictions on data availability

RNA-seq and ChIP-seq data generated in this study were deposited in the GEO database under accession number GSE112697. The data that support the findings of this study are available from the corresponding author upon reasonable request.

Field-specific reporting

Please select the one below that is the best fit for your research. If you are not sure, read the appropriate sections before making your selection.

☒ Life sciences ☐ Behavioural & social sciences ☐ Ecological, evolutionary & environmental sciences

For a reference copy of the document with all sections, see nature.com/documents/nr-reporting-summary-flat.pdf

Life sciences study design

All studies must disclose on these points even when the disclosure is negative.

Sample size	Pilot analyses and previous literature were used to determine sample size.
Data exclusions	All data from experiments in which the positive and/or negative controls worked appropriately were included in the study. If the positive and/or negative controls did not work, the whole experiment was excluded from the analyses.
Replication	All data are from 3-16 individual mice.
Randomization	Genetically modified mice were randomly assigmed to the experiments. Age- and sex-matched wild-types littermates were used as controls.
Blinding	The samples were coded by individual mouse ID code and thus the investigators did not know at the time of experiment what samples were from GM animals and which from controls.

Reporting for specific materials, systems and methods

We require information from authors about some types of materials, experimental systems and methods used in many studies. Here, indicate whether each material, system or method listed is relevant to your study. If you are not sure if a list item applies to your research, read the appropriate section before selecting a response.

Materials & experimental systems

n/a	Involved in the study	
☐	☒	Antibodies
☒	☐	Eukaryotic cell lines
☒	☐	Palaeontology
☐	☒	Animals and other organisms
☒	☐	Human research participants
☒	☐	Clinical data

Methods

n/a	Involved in the study	
☐	☒	ChIP-seq
☒	☐	Flow cytometry
☒	☐	MRI-based neuroimaging

Antibodies

Antibodies used	All antibodies used have been described in Materials and Methods section (Supplier and catalog number)
Validation	The primary antibodies have been validated by the vendors or by our previous published work (Karvonen et al., Interaction of androgen receptors with androgen response element in intact cells. Roles of amino- and carboxyl-terminal regions and the ligand. J. Biol. Chem. 272, 15973–9 (1997))

Animals and other organisms

Policy information about studies involving animals; ARRIVE guidelines recommended for reporting animal research

Laboratory animals	ArKI mice, 129S6BF1;C57Bl/6N hybrid background, male mice at the age range of 2 weeks to 10 months.

Wild animals	The study did not involve wild animals.
Field-collected samples	The study did not involve samples collectd from the field.
Ethics oversight	Animal experiments were approved by the Finnish Animal Ethics Committee, and fully met the requirements of the U.S. National Institutes of Health guidelines on animal experimentation.

Note that full information on the approval of the study protocol must also be provided in the manuscript.

ChIP-seq

Data deposition

☒ Confirm that both raw and final processed data have been deposited in a public database such as GEO.

☒ Confirm that you have deposited or provided access to graph files (e.g. BED files) for the called peaks.

Data access links<br>*May remain private before publication.*	https://www.ncbi.nlm.nih.gov/geo/query/acc.cgi?acc=GSE121151
Files in database submission	GSM3427006 AR-ChIP-seq_WT-Cap_rep1<br>GSM3427007 AR-ChIP-seq_WT-Cap_rep2<br>GSM3427008 AR-ChIP-seq_ArKI-Cap_rep1<br>GSM3427009 AR-ChIP-seq_ArKI-Cap_rep2<br>GSM3427010 Input_WT-Cap<br>GSM3427011 Input_ArKI-Cap
Genome browser session<br>(e.g. UCSC)	no longer applicable.

Methodology

Replicates	Two anti-AR ChIP-seq replicates from WT-AR and ArKI mice were produced. For one sample, caput epididymides (containing initial segments) were pooled from three animals. In WT, replicate 1 had 26266 peaks, replicate 2 had 21151 peaks and there were 18769 common peaks (71% or 89% of peaks. For ArKI, replicate 1 had 24015 peaks, replicate 2 had 38069 peaks, and 23029 peaks were common (96% or 60% of peaks).
Sequencing depth	Data set,Unique reads,Total reads<br>ARKImouse_AR_none_rep1,23129900,23221874.0<br>ARKImouse_AR_none_rep2,29928438,30089467.0<br>ARKImouse_input_none_rep1,25578821,25683505.0<br>wtMouse_AR_none_rep1,22381219,22483872.0<br>wtMouse_AR_none_rep2,25774890,25883953.0<br>wtMouse_input_none_rep1,35772607,35907600.0
Antibodies	Rabbit polyclonal anti-AR antibody K183 (Karvonen et al., 1997 J Biol Chem).
Peak calling parameters	Reads were aligned against mouse genome mm10 with Bowtie software (version 0.12.9) with the command line: -v 1 -k 1 -m 1 -f -S --best mm10. Peak calling was done in HOMER (version 4.10.3) using findPeaks command using default settings (FDR <0.001, fold change > 4) against the input. Fragmented, de-crosslinked chromatin was used as an input.
Data quality	Peaks were called with FDR <0.001.<br><br>Data set,# of peaks,# of peaks with FC > 5,% of peaks with FC > 5<br>wtMouse_AR_none_rep1.pos,26266,25205,96%<br>wtMouse_AR_none_rep2.pos,21151,19579,93%<br>ARKImouse_AR_none_rep1.pos,24015,23016,96%<br>ARKImouse_AR_none_rep2.pos,38069,35236,93%
Software	Overlapping peaks from two replicates was analyzed using bedTools (intersectBed) and used for further analysis. De novo motif analysis was done with HOMER using findMotifsGenome tool with default settings. Heatmap and line profiles of ChIP-seq data were produced using HOMER and visualized using ImageJ and R.
